# Recent Advancements in Mechanistic Studies of Palladium- and Nickel-Catalyzed Ethylene Copolymerization with Polar Monomers

**DOI:** 10.3390/polym15224343

**Published:** 2023-11-07

**Authors:** Zhihui Song, Shaochi Wang, Rong Gao, Ying Wang, Qingqiang Gou, Gang Zheng, Huasheng Feng, Guoqiang Fan, Jingjing Lai

**Affiliations:** 1 Department of Polyethylene, SINOPEC (Beijing) Research Institute of Chemical Industry Co., Ltd., Beijing 100013, China; gaor.bjhy@sinopec.com (R.G.); wangying.bjhy@sinopec.com (Y.W.); gouqq.bjhy@sinopec.com (Q.G.); zhenggang.bjhy@sinopec.com (G.Z.); fangq.bjhy@sinopec.com (G.F.); laijj.bjhy@sinopec.com (J.L.); 2 Department of Chemistry, Vanderbilt University, Nashville, TN 37235, USA; shaochi.wang@vanderbilt.edu; 3 Department of Catalytic Science, SINOPEC (Beijing) Research Institute of Chemical Industry Co., Ltd., Beijing 100013, China; fenghs.bjhy@sinopec.com

**Keywords:** late transition metal catalyst, functionalized polyolefin, copolymerization, coordination polymerization, polar monomer

## Abstract

The introduction of polar functional groups into polyolefin chain structures creates opportunities to enhance specific properties, such as adhesion, dyeability, printability, compatibility, thermal stability, and electrical conductivity, which widen the range of potential applications for these modified materials. Transition metal catalysts, especially late transition metals, have proven to be highly effective in copolymerization processes due to their reduced Lewis acidity and electrophilicity. However, when compared to the significant progress and summary of synthetic methods, there is a distinct lack of a comprehensive summary of mechanistic studies pertaining to the catalytic systems involved in ethylene copolymerization catalyzed by palladium and nickel catalysts. In this review, we have provided a comprehensive summary of the latest developments in mechanistic studies of ethylene copolymerization with polar monomers catalyzed by late-transition-metal complexes. Experimental and computational methods were employed to conduct a detailed investigation of these organic and organometallic systems. It is mainly focused on ligand substitution, changes in binding modes, ethylene/polar monomer insertion, chelate opening, and β-H elimination. Factors that control the catalytic activity, molecular weight, comonomer incorporation ratios, and branch content are analyzed, these include steric repulsions between ligands and monomers, electronic effects arising from both ligands and monomers, and so on.

## 1. Introduction

Polyolefins stand as the predominant plastics, which constitute over half of the global plastics production at 360 million tons and occupy a pivotal role in modern society [1]. Polyolefins possess a wide range of advantages and desirable properties, such as abundant raw materials, cost-effectiveness, chemical stability, corrosion resistance, etc. Despite the escalating worldwide production and demand for polyolefins, the inherent nonpolar nature of their chain structure remains a primary limiting factor, limiting their wider applications in many important fields. The introduction of active polar functional groups into the polyolefin chain structures presents an avenue to enhance surface properties such as adhesion, dyeability, printability, and compatibility, thus broadening the range of potential applications [2,3,4,5,6].

**Figure 1:**
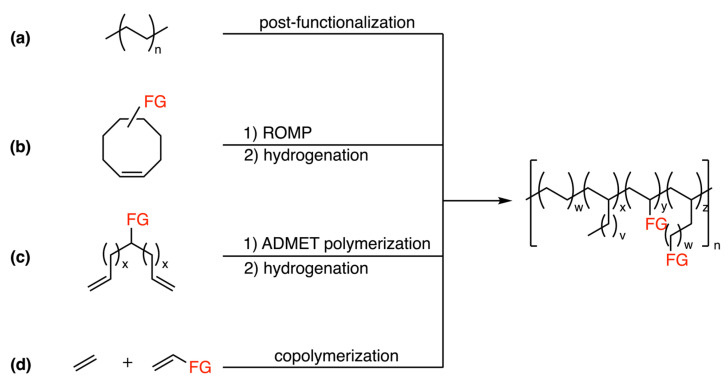
Several synthetic methods for functionalized polyethylene. (**a**) post-functionalization; (**b**) ring-opening metathesis polymerization/hydrogenation; (**c**) acyclic diene metathesis/hydrogenation; (**d**) copolymerization.

Post-functionalization serves as a conventional approach in the synthesis of functionalized polyolefins (Figure 1a) [7]. However, due to the inert quality of the C-H bonds within polymers, these experiments need high temperature and high pressure, resulting in side-reactions triggered by radical recombination. Strategies like ring-opening metathesis polymerization (ROMP) (Figure 1b) [8,9,10] and acyclic diene metathesis (ADMET) (Figure 1c) [11,12,13,14], followed by hydrogenation, are frequently employed for the preparation of functionalized polyolefins. Nevertheless, specific monomers are required for these multi-step reactions, thereby increasing the associated costs.

**Figure 2:**
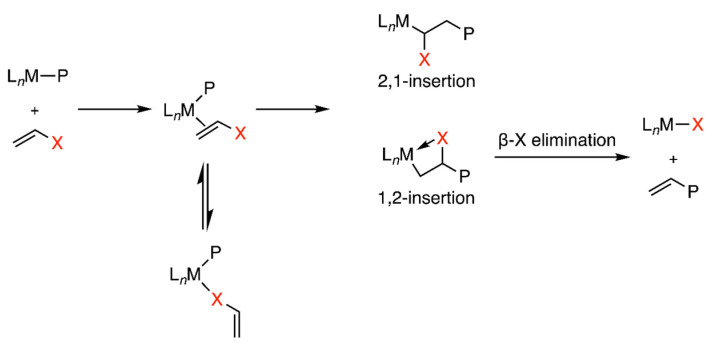
Mechanism for transition-metal-catalyzed ethylene copolymerization.

Alternatively, coordination polymerization, especially transition-metal-catalyzed copolymerization emerges as a straightforward and cost-effective approach to precisely regulate polar/nonpolar ratios and polymer microstructures under mild conditions (Figure 1d) [15]. However, early transition metal complexes such as Ziegler-Natta catalysts and metallocene-based catalysts face limitations in ethylene copolymerization [16]. These deficiencies mainly arise from the deactivation of the Lewis acidic metal center due to interaction with Lewis basic polar groups, typically containing oxygen and nitrogen. This interaction leads to the formation of stable metal-alkyl chelates, followed by termination steps such as β-X elimination (Figure 2).

To address the challenge posed by polar monomers in Figure 2, late transition metal catalysts, including palladium and nickel complexes, were employed. Pd- and Ni-based catalysts exhibit better tolerance for functional/polar groups under mild reaction conditions, primarily due to their lower Lewis acidity, lower electrophilicity, and lower oxophilicity when compared to early transition metal complexes. These properties result in enhanced reactivity when engaging in olefin copolymerization with polar monomers.

**Figure 3:**
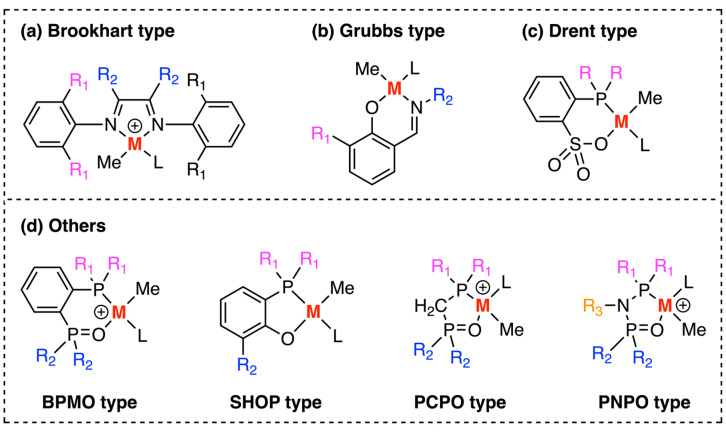
Several typical late transition metal catalysts. (**a**) Brookhart catalyst; (**b**) Grubbs catalyst; (**c**) Drent catalyst; (**d**) other catalysts.

In 1995, Brookhart and co-workers reported the α-diimine palladium and nickel complexes (Brookhart catalyst, Figure 3a) could catalyze ethylene polymerization with high molecular weight [17]. Importantly, the Brookhart palladium catalyst could enable the olefin copolymerization with methyl acrylate, making it a great breakthrough in this field. Later, other esters and silyl vinyl ethers were found suitable for copolymerization with this type of catalyst.

In 2000, Grubbs and co-workers discovered the neutral nickel(II) salicylaldimine catalysts (Grubbs catalyst, Figure 3b) facilitated copolymerization between ethylene and substituted norbornenes with no co-catalyst [18]. It also showed the influence (activity, molecular weight, and the degree of branch) made by the bulkiness of the substituent adjacent to the O atom. In 2002, Drent and colleagues reported a series of phosphine-sulfonate palladium complexes (Drent catalyst, Figure 3c) could catalyze ethylene copolymerization with alkyl acrylates [19]. Subsequently, Mecking [20] and Chen [21] tried different substituents on the P atom, improving reaction activity and catalyst stability.

Apart from the catalysts above, other scientists have also developed analogous electron-rich complexes based on late transition metals. Notable examples are from reports by Nozaki (BPMO type and PCPO type) [22,23], Shimizu (SHOP type) [24], and Chen (PNPO type) [25]. They used Pd- or Ni-based complexes with nucleophilic ligands to perform olefin copolymerization with various polar monomers such as alkyl acrylates and vinyl ethers (Figure 3d).

With the rapid development in this area, the mechanistic study of these catalytic copolymerization systems has become indispensable. Given the inherent complexity of various catalytic systems, the detailed factors that control activity, molecular weight, comonomer incorporation ratios, and branch content in these transformations remain being explored. To advance the late transition-metal-catalyzed olefin copolymerization, many aspects of mechanistic investigation were considered, such as electronic inhibition, steric inhibition, metal center, etc.

In this comprehensive review, we first focus on the methodologies used conventionally to investigate the mechanisms of olefin polymerizations. Subsequently, computational advancements in olefin copolymerization employing Brookhart [26,27,28,29,30,31,32,33,34], Grubbs [27,28,35,36,37,38,39], Drent [34,40,41,42,43,44,45,46], and other catalytic systems [47,48,49,50,51,52,53,54,55,56,57] will be discussed. Specifically, we concentrate on the essential steps of different types of polar monomers. Finally, based on this study, we summarize the reaction steps, shed light on the factors that influence the results of the copolymerization from the view of the mechanistic study, and provide a blueprint for the rational design of relevant catalysts and transformations.

## 2. Experimental and Computational Methods to Investigate the Mechanisms

In this review, the mechanistic study has benefited from experimental methods developed to study the organic and organometallic systems. X-ray crystallography emerges as a powerful tool to elucidate the conformations of organometallic species, including both intermediates and catalysts [31,33,47,48]. Electron paramagnetic resonance (EPR) spectroscopy is commonly used to detect the paramagnetic shifts exhibited by organic and organometallic species throughout the polymerization process [39]. From the EPR spectra, changes in the chemical shift can indicate the presence of unpaired electrons, revealing the formation of radicals at particular steps in the catalytic process. NMR spectroscopy, especially low-temperature NMR experiments could be applied to track the reactions and provide insights into the kinetic behavior [31,32,33,36,37,38,39,41,42,47,48]. Low-temperature experiments can produce active species throughout the reaction process, and these findings can be elucidated through NMR analysis. The use of control experiments in conjunction with NMR analysis could clarify the intermediates involved in the reactions. Additional experimental methodologies such as IR [33], and GC-MS [33,36] have been employed to unravel the details of the experiments, contributing to a comprehensive understanding of the polymerizations.

Quantum mechanical calculations were used to unravel the mechanisms and design novel catalysts. These calculations mainly employed density functional theory (DFT) due to its balance between computational cost and chemical accuracy [58,59,60,61,62,63,64,65,66,67]. DFT calculations cited in this review were executed through various platforms, including Amsterdam density functional (ADF) [26,27,28,29,30,34,35,40,68], Gaussian03 [41,42,69], Gaussian09 [33,43,44,49,50,51,53,70], and Gaussian16 [45,46,48,52,53,54,55,56,57,71]. The details of DFT calculations such as functionals, basis sets, and implicit solvents are based on the type of catalysts, experimental conditions, and the preference of the computational chemists. Notably, molecular dynamics (MD) [29] and quantum mechanics/molecular mechanics (QM/MM) [30,34,40] were utilized to explore the intrinsic polymerization process from a theoretical perspective.

## 3. Examples of Mechanistic Studies of Palladium and Nickel-Catalyzed Ethylene Polymerizations

### 3.1. Brookhart-Type Catalysts

**Figure 4:**
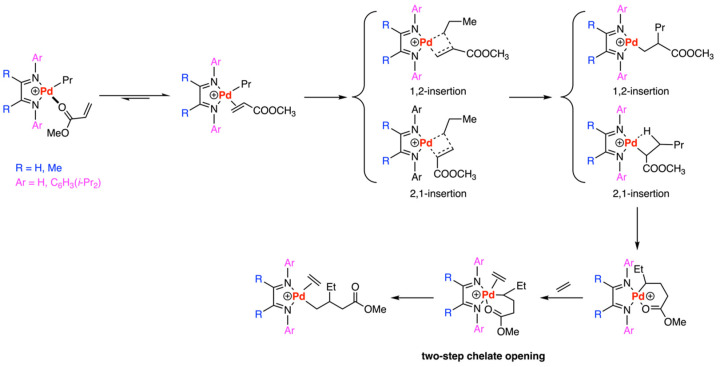
Pd-diimine-catalyzed copolymerization between ethylene and methyl acrylate (MA).

In 2001, Ziegler and his colleagues [26] employed density functional theory (DFT) to investigate the fundamental reaction steps in the copolymerization of ethylene with methyl acrylate (MA) catalyzed by Pd-diimine catalysts (Figure 4). The stability of the acrylate complexes were discussed, resulting in the π-complex being energetically preferred by 3.4 kcal/mol over the O(carbonyl)-complex. Subsequently, 1,2- and 2,1-insertion of MA were examined. For the generic catalyst (R = H, Ar = H), the transition state of 2,1-insertion exhibited a substantial preference, being lower in energy by 4.5 kcal/mol compared to that of 1,2-insertion. However, for the bulkier real system (R = Me, Ar = C_6_H_3_(*i*-Pr_2_)), 2,1-insertion has a lower barrier of approximately 0.5 kcal/mol compared to 1,2-insertion. The energetic difference arises from the steric repulsion between the olefin substituent (-COOCH_3_) and the alkyl chain, which destabilizes the transition state of 1,2-insertion rather than 2,1-insertion. Following 2,1-insertion and isomerization, the six-membered-ring chelate emerged as the most stable insertion product, with an energy advantage of 21 kcal/mol over the β-agostic 2,1-insertion product. In turn, the chelate-opening involving ethylene and MA was studied. Due to the higher barrier associated with the opening of the six-membered-ring chelate and the steric effects of the real ligand, the reaction suggests the possibility of a two-step chelate-opening mechanism with a lower barrier.

**Figure 5:**
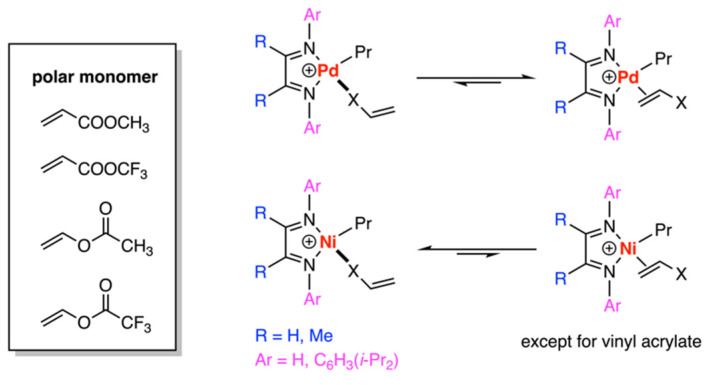
Pd-diimine-catalyzed copolymerization between α-olefins and polar monomers.

In the same year, Ziegler and his research team also conducted DFT calculations regarding the copolymerization of α-olefins with polar monomers [27]. Their investigation concentrated on the binding modes of comonomers, such as methyl acrylate, vinyl acetate, and their fluorinated derivatives, with nickel- and palladium-based Brookhart (cationic) ligands (Figure 5) and Grubbs ligands (neutral) (Figure 8). For the Pd-based Brookhart system, the π-complex proved to be more stable than the corresponding O(carbonyl)-complexes by 3.4–6.7 kcal/mol. However, in the case of the Ni-diimine system, O(carbonyl)-complexes were generally preferred, with the exception of fluorinated vinyl acrylate which aligns with the inactive Ni system. Moreover, the fluorinated monomers could destabilize the O(carbonyl)-complexes and thereby change the binding mode preference of the Ni-diimine system. Nevertheless, this change also affected the stabilization of the π-complexes, resulting in a reduced incorporation of the polar comonomer. The use of fewer oxophilic catalysts (Grubbs type) was studied and will be mentioned in Section 3.2. Nitrogen-containing polar monomers [CH_2_=CH(CH_2_)_n_X (n ≥ 0; X = CN, NH_2_, N(CH_3_)_2_)] have been studied by the same group and π-complexes with the α-diimine ligand are also favored over that of N-complexes [28].

Apart from the investigations into the polar monomer binding mode above [27,28], both DFT and MD theoretical study has been used to investigate the reasons for the distinct reactivities exhibited by Pd-(active catalyst) and Ni-(inactive under the same conditions) α-diimine complexes in the copolymerization of ethylene and methyl acrylate (MA) [29]. Both systems were found to follow similar mechanisms that π-complexation, MA 2,1-insertion, chelate opening, ethylene complexation, and insertion, as previously reported in (Figure 4) [26]. For the real diimine catalyst, the barriers of MA insertion with Ni and Pd complexes were comparable (13.5 kcal/mol vs. 12.4 kcal/mol). In addition, the barriers for the MA insertion were lower than those of ethylene insertion (16.8 and 14.2 kcal/mol for Pd- and Ni-catalyst, respectively). Consequently, MA insertion and ethylene insertion could not account for the difference in the copolymerization with Pd- and Ni- complexes. After the formation of chelates from the MA insertion, although the endothermicity of chelate-opening before the ethylene reinsertion is higher for Ni- than for the Pd-system (10.9 and 6.8 kcal/mol, respectively) for the genetic catalysts (R = H, Ar = H), the steric repulsion-induced substrates on the catalyst (Ar = 2,6-C_6_H_3_(*i*-Pr)_2_; R = Me) facilitates the chelate-opening and this effect is stronger for nickel than for palladium with the endothermicity is 1.8 kcal/mol for Ni and 2.5 kcal/mol for Pd. Thus, the chelate-opening step is not the key to the different reactivities, and the steric bulk is likely to weaken the chelate bonds that might poison the catalyst. Finally, the results suggest that the initial poisoning of the catalyst by the O-binding mode in the case of the Ni system, as opposed to the lower energy π-complex in the Pd-system, is the key factor driving the different reactivities between Pd and Ni complexes.

**Figure 6:**
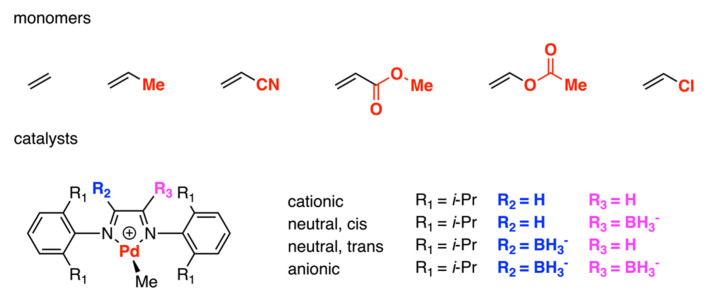
Pd−diimine−catalyzed copolymerization between ethylene and CH_2_=CHX monomers.

Later, in 2004, DFT and QM/MM theoretical comprehensive studies were used to explore the molecular-level understanding of the Pd-diimine-catalyzed copolymerization between ethylene and the CH_2_=CHX monomers (where X = -H, -Me, -CN, -COOMe, -OC(O)Me, -Cl) (Figure 6) [30]. The cationic diimine complex and its modified neutral and anionic derivatives have also been investigated. Regarding the binding mode to the metal center, π-complexation was more favorable than σ-complexation for all monomers with all ligands, except for acrylonitrile bound to the cationic complex. Considering the π-donation to the metal, the π-complexation is strongest for propylene and weakest for acrylonitrile, with the oxygen-containing polar monomers and vinyl chloride in between. In addition, since the destabilization is effective on both π-complex and the relevant transition states, polar group X has only a minor effect on the step of CH_2_=CHX insertion into the Pd-Me bond. Moreover, when the ligand became neutral and negative, the insertion barriers were found to be increased. Meanwhile, the barrier for insertion of ethylene into the Pd-CH(X)CH_2_CH_3_ bond was found to be larger with the increase in the electron-withdrawing ability of the X substituents and the electron charge of the ligand, partly due to the stability of the chelate.

**Figure 7:**
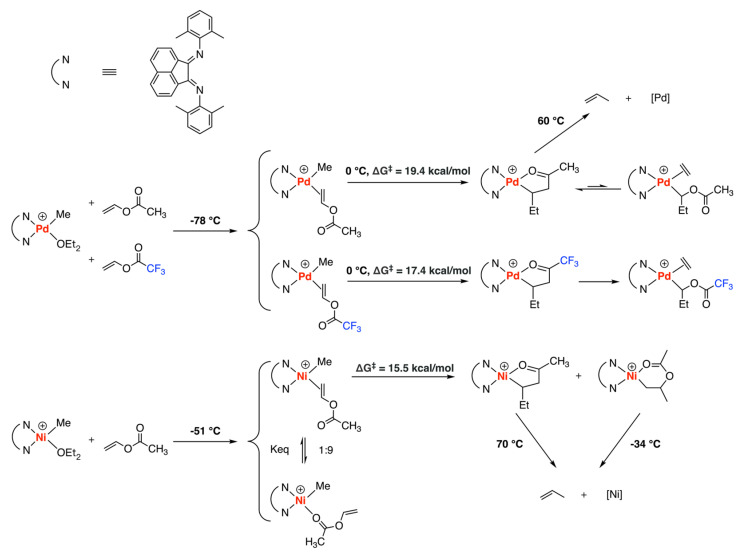
Ni− and Pd− diimine catalyzed ethylene copolymerization with VA/VAf.

In 2005, Brookhart and coworkers reported a detailed experimental mechanistic study of the ethylene copolymerization with vinyl acetate (VA)/vinyl trifluoroacetate (VAf) using Ni- and Pd-diimine complexes as catalysts (Figure 7) [31]. Low-temperature experiments revealed the Pd system preferred π-coordination with monomer and underwent 2,1-insertion to form 5-membered-ring chelate which was confirmed by NMR and XRD, both were used to identify the species in the catalytic system. According to the Eyring Plot, the barrier of VA insertion (19.4 kcal/mol) was larger than that of VAf insertion (17.4 kcal/mol), owing to the increased electrophilicity of the olefinic group from the electron-withdrawing group trifluoroacetoxy. However, the nickel system exhibited a preference for O-complexation over π-complexation with a ratio of 9:1, which aligned with previous predictions [27,29]. Considering the equilibrium between O-complex and π-complex, the estimated barrier for VA insertion was 15.5 kcal/mol, and for VAf insertion, it was 17.3 kcal/mol. Thermal analysis experiments were conducted to assess the stability of the insertion products, revealing that β-acetate and β-trifluoroacetate elimination occurred, which suggested that the stability of nickel complexes was slightly greater than the analogous palladium species, possibly due to the stronger chelation to the oxophilic nickel center. Additionally, it was noted that the five-membered chelate resulting from VAf insertion could be opened by ethylene to form the ethylene adduct, while the chelate formed from VA insertion exhibited a preference for chelation over ethylene adduct formation. Furthermore, the barriers for ethylene reinsertion into the opened forms of the VA and VAf chelates were significantly higher (approximately 3 kcal/mol) than the barrier for ethylene insertion into the (Pd-alkyl)^+^ bond due to the electron-withdrawing group (acetoxy and trifluoroacetoxy groups) at the α-carbon.

**Figure 8:**
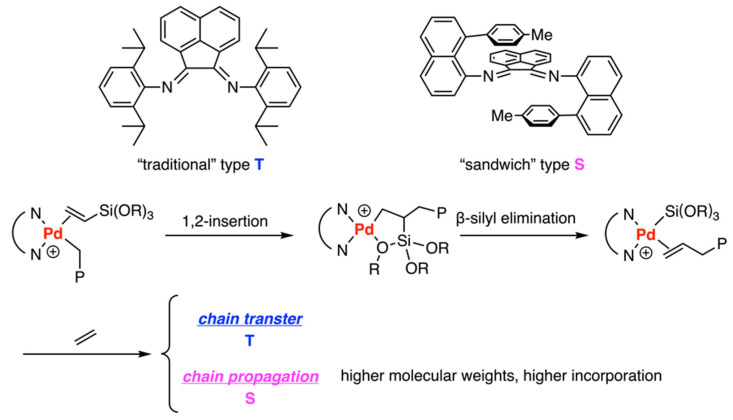
Copolymerization of ethylene and vinylalkoxysilanes catalyzed by “traditional” type catalyst and “sandwich” type catalyst.

In 2016, low-temperature NMR mechanistic studies by Brookhart and coworkers were performed to delve into the molecular-level understanding of the copolymerization of ethylene and vinylalkoxysilanes catalyzed by a “traditional” Pd(II) aryldiimine catalyst (aryl = 2,6-diisopropylpheny) and a “sandwich” aryldiimine catalyst (aryl = 8-tolylnaphthyl) (Figure 8) [32]. Meanwhile, Eyring plots were used to obtain the barrier (ΔG^‡^) and gain insights into the reaction mechanisms. For both catalysts, migratory insertion of the vinylalkoxysilane underwent 1,2-insertion selectively to form five-membered chelates. Interestingly, the barriers of vinylalkoxysilanes insertion were slightly higher (less than 1 kcal/mol) than those for ethylene insertions for both ligands. Chain transfer via β-silyl elimination allowed the formation of low molecular weight copolymers in the traditional catalyst system but was retarded in the sandwich system, which interpreted that the sandwich system enjoys higher molecular weight. The relative binding affinities of vinyltrialkoxysilanes and ethylene were identified as key factors in determining the incorporation ratio of the comonomer. The sandwich system exhibited a higher binding affinity with vinyltrialkoxysilanes compared to the traditional system, which leads to a higher incorporation ratio of the sandwich system.

**Figure 9:**
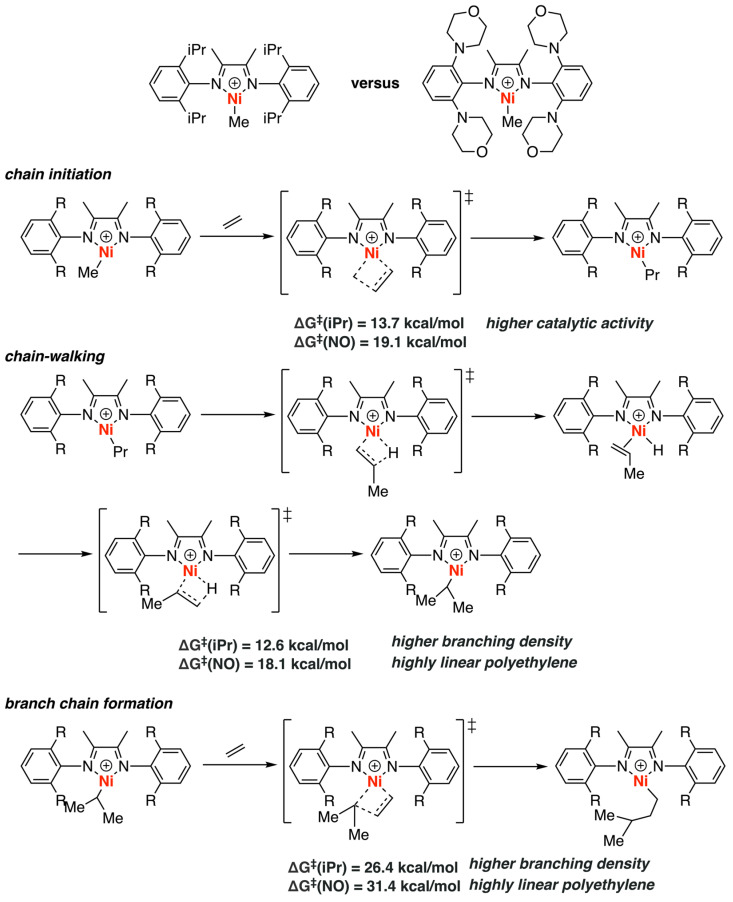
Comparisons among different types of α-diimine catalysts.

In 2017, Chen and Luo investigated the properties of the nitrogen-containing α-diimine-based nickel and palladium catalysts in ethylene homopolymerizations and copolymerizations by a combination of experimental (NMR) and computational (DFT) methods (Figure 9) [33]. Their study aimed to understand how different ligands and metal centers affect the polymerization processes. The homopolymerization processes of ethylene catalyzed by two different α-diimine ligands (isopropyl as well as nitrogen-containing groups on aryl moiety) of nickel complexes were compared through theoretical calculations. The entire polymerization process includes first molecule ethylene insertion (chain initiation), β-H elimination followed by ethylene reinsertion (chain-walking), and branched chain formation (chain propagation). Among them, the energy barrier for chain initiation with a nitrogen-containing nickel catalyst is 19.1 kcal/mol, higher than that of isopropyl by 5.4 kcal/mol, which is consistent with the experimental results that the nitrogen-containing α-diimine-based nickel complex has lower catalytic activity compared to that of isopropyl. Additionally, the nitrogen-containing nickel complexes also have higher barriers for chain-walking and branched chain formation steps, which explains the lower branching density compared to the traditional isopropyl-containing catalyst. Apart from nickel catalysts, the researchers also investigated the energy profiles for palladium-catalyzed ethylene-allyl chloride copolymerizations using computational methods. The results showed a much higher barrier for β-Cl elimination in the case of nitrogen-containing palladium catalysts compared to the traditional isopropyl-containing Brookhart catalyst because the Pd-N interaction may partly block the coordination of the Cl atom to the Pd center, which rationalizes that the nitrogen-containing palladium catalysts could catalyze the copolymerization between ethylene and allyl chloride. These results revealed the key role of the secondary coordination of metal to nitrogen in these polymerizations.

### 3.2. Grubbs-Type Catalysts

**Figure 10:**
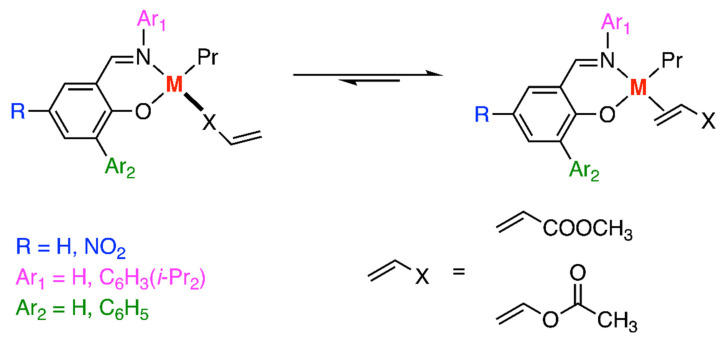
Copolymerization of α-olefins with oxygen-containing monomers catalyzed by nickel- and palladium-based Grubbs complexes.

In 2001, Ziegler and coworkers also used a DFT study to explore the copolymerization of α-olefins with oxygen-containing monomers, specifically methyl acrylate (MA) and vinyl acetate (VA), catalyzed by nickel- and palladium-based Grubbs (neutral) ligands (Figure 10) [27]. For both Ni- and Pd-based Grubbs complexes, the π-complexes were strongly preferred over the corresponding O(carbonyl)-complexes. Furthermore, the introduction of bulky substituents on the phenyl ring of the Grubbs catalysts led to a decrease in the binding energies of both the π- and O(carbonyl)-complexes. However, this change in binding energy did not affect the overall preference for the binding mode. These findings clarified that the polar monomers are bounded by their olefinic functionality rather than the carbonyl oxygen for the less oxophilic system. Nitrogen-containing polar monomers [CH_2_=CH(CH_2_)_n_X (n ≥ 0; X = CN, NH_2_, N(CH_3_)_2_)] were investigated in the following year, revealing that π-complexes with nickel- and palladium-based salicylaldiminato ligand are also preferred over that of N-complexes [28].

**Figure 11:**
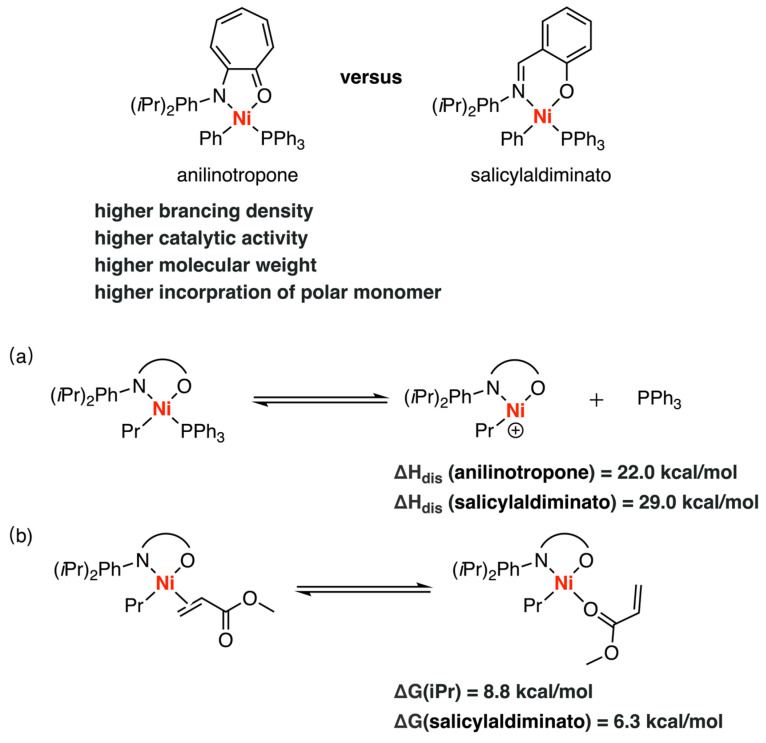
Comparison of ethylene copolymerization catalyzed by (**a**) Ni-anilinotropone complex and (**b**) Ni-salicylaldiminato complex.

In 2003, Ziegler et al. investigated the mechanisms of ethylene polymerization catalyzed by the Ni-anilinotropone and Ni-salicylaldiminato complexes by DFT calculations [35]. Phosphine dissociation, chain propagation/isomerization, and the methyl acrylate binding modes have been investigated to understand why the Ni-anilinotropone catalyst could possess better functional group tolerance, higher catalytic activity, and higher molecular weight product compared to the Ni-salicylaldiminato catalyst (Figure 11) [72,73]. The results revealed that, for the anilinotropone complex, the phosphine dissociation required less energy (22 kcal/mol) compared to the salicylaldiminato system (29 kcal/mol), supporting the corresponding experimental hypothesis [72,73]. Moreover, the anilinotropone complex favored energetically favorable branched alkyl agostic complexes over less branched and linear isomers, resulting in the formation of branched products. Additionally, the anilinotropone complex exhibited a greater energy preference (8–13 kcal/mol) for π-complexes over O(carbonyl)-complexes, whereas the Grubbs catalyst showed a smaller preference (6.3 kcal/mol), rationalizing the high functional group tolerance of the anilinotropone complex. Furthermore, the results indicated that the steric effects caused by bulky substrates had a lesser impact on the acrylate π-complexation energies in the anilinotropone system compared to salicylamide catalysts, leading to a higher incorporation of polar monomers.

**Figure 12:**
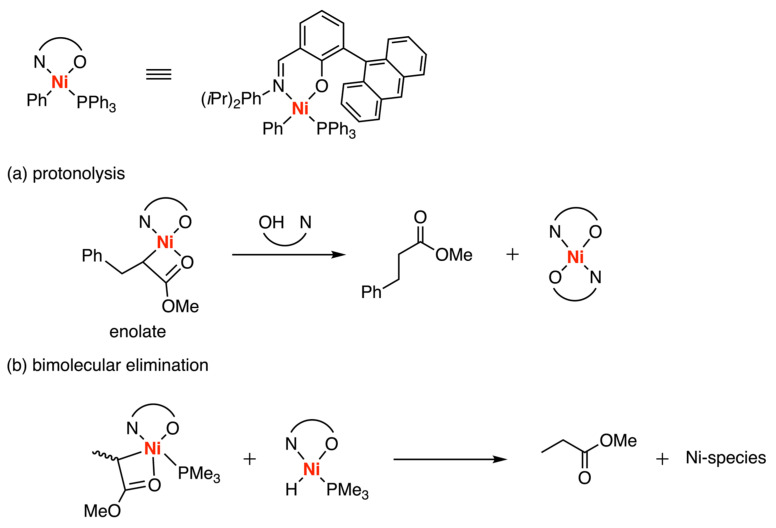
Decomposition pathways of nickel-based Grubbs complex with functionalized olefins. (**a**) protonolysis; (**b**) bimolecular elimination.

In 2004, the Grubbs group used experimental methods NMR and GC-MS to study the deactivation of nickel-based Grubbs complex in the presence of functionalized olefins, including methyl acrylate (MA) and analogous acrylates (Figure 12a) [36]. Their findings revealed the initial insertion of MA resulted in the formation of a highly active nickel enolate complex, which exhibited greater susceptibility to protonolysis compared to the nonchelating alkyl group derived from nonpolar monomers. This protonation was believed to be initiated by the free ligand, salicylaldimine, generated through the reductive elimination of Ni-hydride species. Subsequently, the Mecking group reported a comprehensive mechanistic study of neutral Ni(II) salicylaldimine catalyst insertion products of MA and VA by low-temperature NMR spectroscopic observations [37,38], showing an additional decomposition pathway involving bimolecular elimination with Ni-hydride species (Figure 12b).

**Figure 13:**
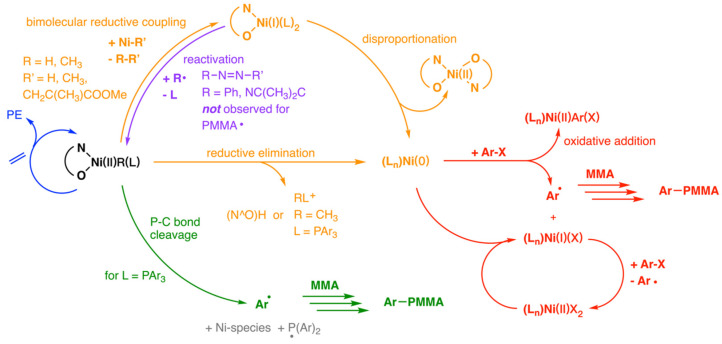
Overview of the mechanism of the Ni(II)−salicylaldiminato system in the presence of monomers.

In 2015, the Mecking group employed NMR, EPR, and other experimental methods to investigate the migratory insertion polymerization of ethylene in the presence of methyl methacrylate (MMA) using Ni(II)-salicylaldiminato complexes, revealing an interlocking mechanistic that possible formation pathways and multiple roles of radicals in Ni(II) catalyzed polymerizations (Figure 13) [39]. First, the ethylene polymerization follows a migratory insertion pathway (Figure 13, blue) which remained unaffected by the radical polymerization of MMA. Then, it was observed that the insertion products Ni-alkyl complexes of MMA into the Ni-H and Ni-Ph complexes were thermally unstable and tended to undergo decomposition either through reductive elimination or bimolecular reductive coupling, leading to the formation of Ni(0) complex. In addition, a homolytic P-C bond cleavage of aryl phosphine ligands (Figure 13, green) could generate the aryl radical at the end of the polymer chain. Meanwhile, one electron oxidation/single electron transfer of Ni(0) species served as the second radical source, followed by polymerization with MMA to form the PMMA chain (Figure 13, red). Moreover, intermediately formed Ni(I) species were found to potentially transform back into the Ni(II) (aryl/alkyl) complexes rather than undergoing disproportionation into Ni(0) complexes (Figure 13, purple).

### 3.3. Drent Type Catalysts

**Figure 14:**
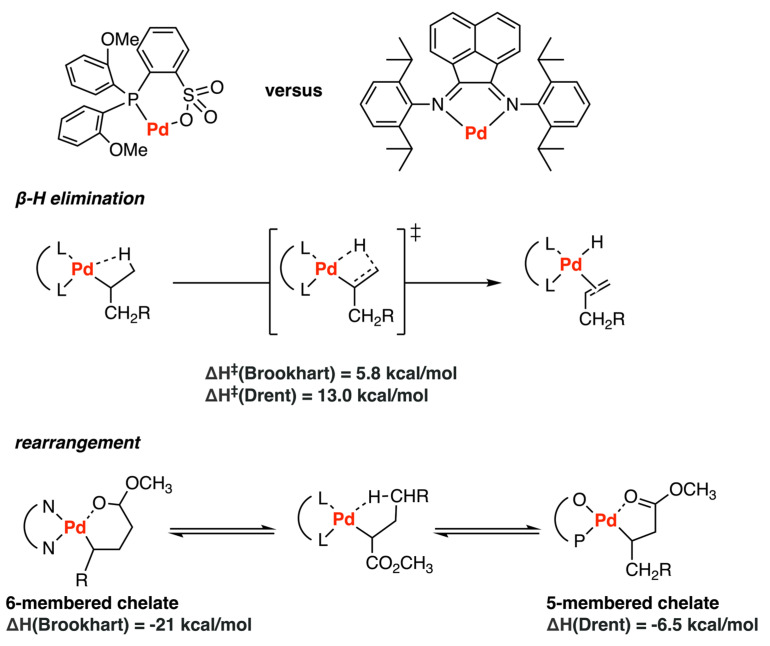
Comparison of ethylene copolymerization catalyzed by Pd−based phosphinate sulfonate complex and α−diimine complex.

In 2006, the mechanisms of ethylene homopolymerization and copolymerization with neutral palladium phosphinate sulfonate complexes (Drent-type catalysts) were investigated by Ziegler et al. [40] with a DFT study and were compared with the counterpart of cationic palladium α-diimine complexes (Brookhart-type catalysts) [34] (Figure 14). For the ethylene homopolymerization, the ethylene complexation energies and the barriers for ethylene insertion into the Pd-alkyl bond were similar in both systems and agreed with the π-complexes as resting states. The great energetic difference between the barriers for β-hydride elimination (~13 kcal/mol for the Drent system and ~6 kcal/mol for the Brookhart system) explained the extensive branching density in the diimine system and the linear polymers in the phosphinate sulfonate system. Furthermore, the thermodynamic six-membered-ring product was energetically favored over the kinetic γ-agostic product by approximately 21 kcal/mol for the α-diimine system, while the thermodynamic five-membered chelate was found to be more stable than the kinetic product by 6.5 kcal/mol for the phosphinate sulfonate system, making it more feasible for the subsequent propagation step. These results were consistent with the experimental observations of low incorporation of MA into a linear polyethylene chain in the Drent system.

**Figure 15:**
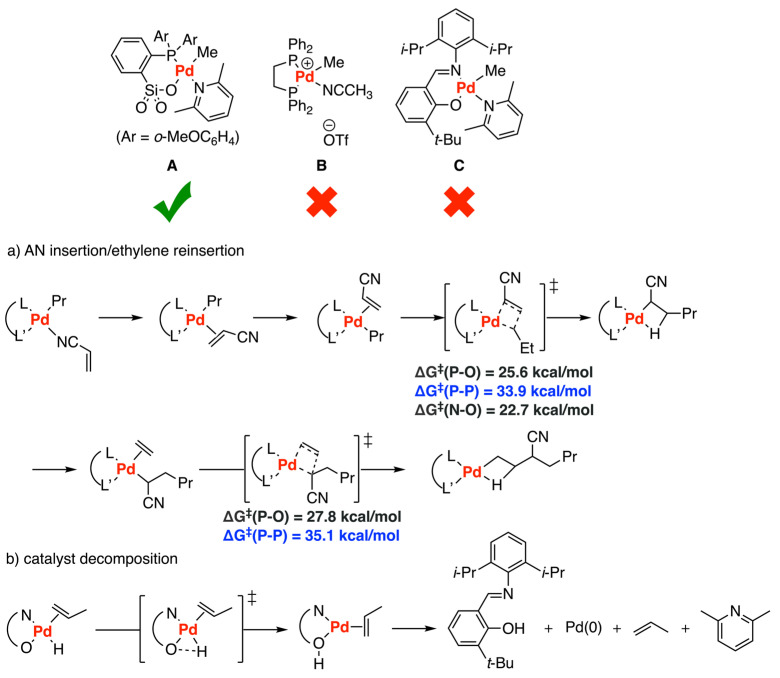
Ethylene copolymerization catalyzed by Pd-based phosphinate sulfonate **A**, diphosphine **B**, and imine-phenolate complex **C**. (**a**) AN insertion/ethylene reinsertion (**b**) catalyst decomposition.

Apart from the homopolymerization of ethylene catalyzed by palladium phosphinate sulfonate catalysts [41], Nozaki and colleagues performed detailed DFT calculations to explore the mechanism of ethylene/acrylonitrile (AN) copolymerization on the key steps including ethylene insertion, acrylonitrile insertion, β-H elimination, chain transfer, and catalyst decomposition with 3 catalysts **A**–**C** [42] (Figure 15). According to the previous study, the copolymerization of ethylene with AN could be catalyzed only by complex **A** instead of **B** or **C** [74]. By comparing phosphine-sulfonate complex **A** and diphosphine complex **B**, it was elucidated that the π-complex [(L–L′)PdPr(π-AN)] is less stable than the corresponding σ-complex [(L–L′)PdPr(σ-AN)] for both systems. Moreover, the energetic difference between the π-complex and the σ-complex is smaller for the phosphine-sulfonate **A** system than the diphosphine **B** system, which leads to the barriers of the transition states for both AN insertion and subsequently ethylene insertion for **A** are lower than that of **B**. In addition, no large difference was found in both AN insertion and the following ethylene insertion between the phosphine sulfonate **A** system and the imine phenolate **C** system. However, for the **C** complex, relevant experiments revealed that β-hydride elimination terminated the polymerization and produced the Pd-H species which could undergo reductive elimination to form free ligand and Pd(0) particles, ending the reaction in catalyst decomposition. Finally, the incorporation ratio and chain-end structure of the copolymer obtained by catalyst **A** could be estimated by using the values they calculated.

**Figure 16:**
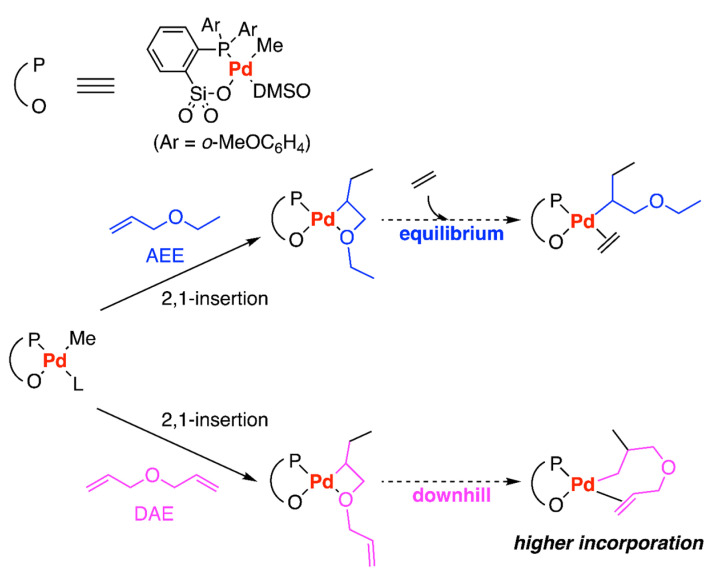
Pd-catalyzed ethylene copolymerization with AEE and DAE.

In 2018, Falivene and colleagues utilized DFT calculations to explore the comparison of ethylene copolymerization with allyl ethyl ether (AEE) and diallyl ether (DAE) (Figure 16) [43]. For both comonomers, the 2,1-insertion was slighter favored compared to the 1,2-insertion kinetically, and the stable five-membered ring with O-chelate coordination from 1,2-insertion is the main obstacle to the chain propagation, therefore leads to the 2,1-insertion be the major mode in the copolymerization. The main difference between these two systems is the subsequent ethylene insertion. For the diallyl monomer, after the first DAE 2,1-insertion, the coordination and insertion of the second monomer were feasible, leading to the formation of a Pd-alkyl complex with β-agostic interaction. This intermediate was likely to be broken by ethylene coordination insertion, which was energetically downhill by approximately 10 kcal/mol. However, for the AEE system, the ethylene coordination intermediate is in equilibrium with the 2,1-insertion O-chelate product. These computational results accounted for the experimental results that the ethylene copolymerization with AEE is challenging while that of DAE is achievable with higher productivity and higher incorporation (4 mol % vs. 20 mol %) [75].

**Figure 17:**
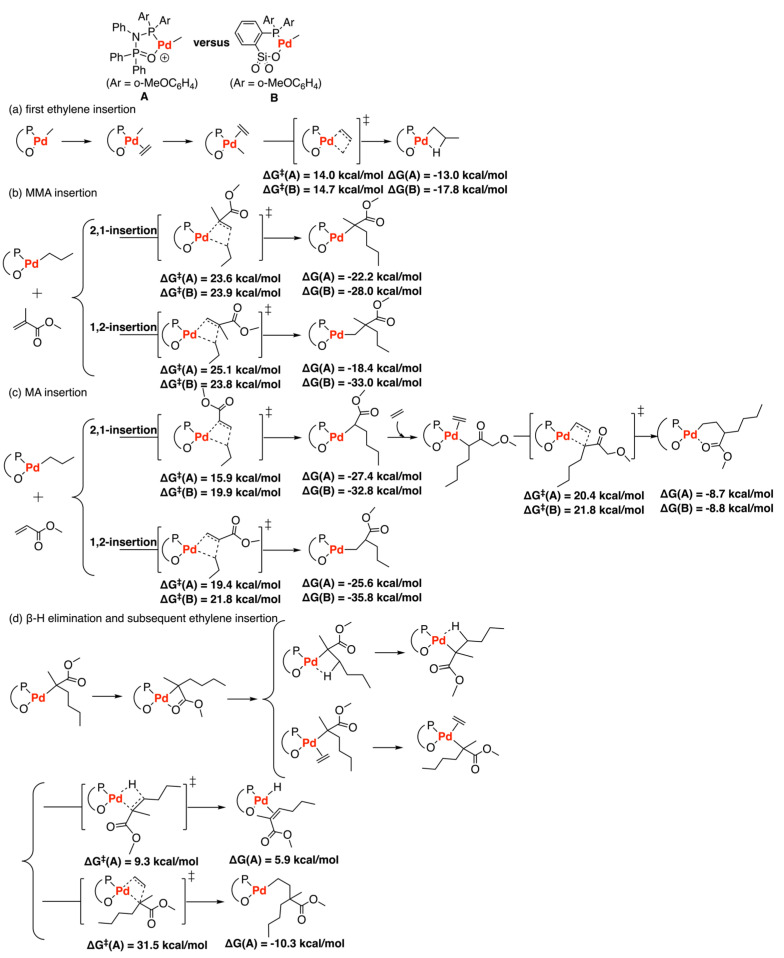
Copolymerization of ethylene with methyl acrylate (MA) and methyl methacrylate (MMA) catalyzed by PNPO−type (**A**) and Drent−type (**B**) palladium catalysts. (**a**) first ethylene insertion; (**b**) MMA insertion; (**c**) MA insertion; (**d**) β−H elimination and subsequent ethylene insertion.

In 2019, DFT calculations were carried out to investigate the copolymerization of ethylene with methyl acrylate (MA) and methyl methacrylate (MMA) by two different palladium catalysts: diphosphazanemonoxide (PNPO-type, **A**) and phosphine-sulfonate (Drent-type, **B**) complexes (Figure 17) [44]. According to a previous study [76], complex **A** could incorporate MMA into ethylene copolymerization with an MMA unit only at the chain end, while complex **B** could not catalyze this copolymerization. Also, experimental results showed that complex **B** could undergo both 1,2- and 2,1-insertions of MMA, while complex **A** is favorable to 2,1-insertion of MMA [25]. Because the steric factor (geometrical deformation) and the stronger MeO…H interaction leads to the higher stability of the 2,1-insertion TS of MMA, the 2,1-insertion mode in the diphosphazane-monoxide **A** system is preferred to 1,2-insertion (barrier is 23.6 kcal/mol vs. 25.1 kcal/mol, respectively). In addition, for the ethylene copolymerization with MA, the rate-determining step is found to be the ethylene reinsertion after MA insertion rather than the MA insertion itself. The barrier of the rate-determining step for system **B** is 21.8 kcal/mol while that of the diphosphazane-monoxide **A** system is 20.4 kcal/mol due to the rigidity of the five-membered backbone. Furthermore, the β-H elimination step has a feasible barrier (9.3 kcal/mol) compared with the barrier of subsequent ethylene insertion (31.5 kcal/mol), which results in the chain-end MMA in the copolymer.

**Figure 18:**
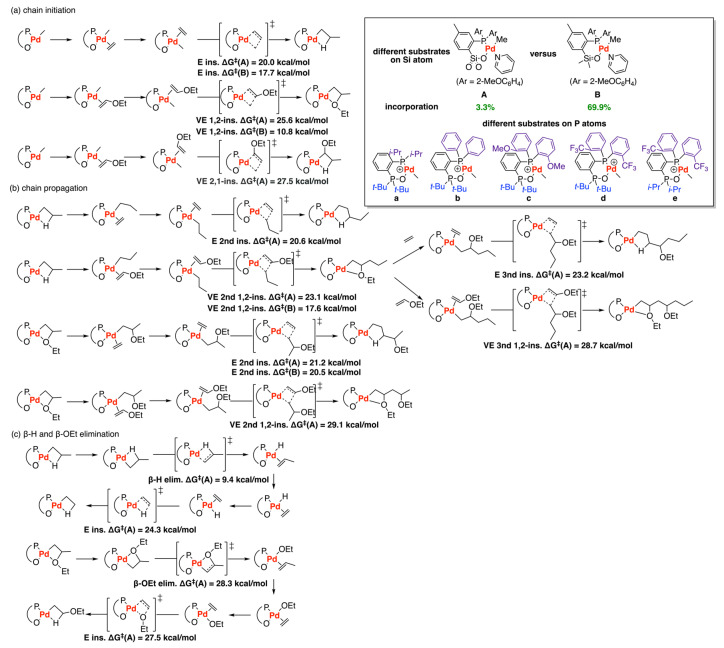
Ethylene copolymerization with vinyl ether (VE) copolymerization catalyzed by palladium phosphine-sulfonate complex (**A**) and (**B**). (**a**) chain initiation; (**b**) chain propagation; (**c**) β−H and β−OEt elimination.

In 2020, DFT calculations were used to investigate the mechanism of ethylene copolymerization with vinyl ether (VE) catalyzed by palladium phosphine-sulfonate complex (**A** and **B**) (Figure 18) [45]. Their study focused on chain initiation, chain propagation, and chain termination (β-H elimination and β-OEt elimination) steps. The calculations revealed that VE 1,2-insertion was more favorable than VE 2,1-insertion, which may be due to the stronger hydrogen bond between the oxygen atom of VE and the ancillary ligand of catalyst **A**. In the chain initiation step, the Boltzmann equation within relevant barriers (20.0 kcal/mol vs. 25.6 kcal/mol) suggested that the VE 1,2-insertion has a 3.3% probability and the ethylene insertion has 96.7% probability, which is in agreement with the experimental results (VE incorp. 3.5%) [77]. After ethylene insertion, the subsequent VE insertion has a higher barrier of 23.1 kcal/mol than that of ethylene insertion (20.6 kcal/mol), indicating lower catalytic activity for ethylene copolymerization with VE compared to ethylene homopolymerization. After VE insertion into the Pd-Me complex, ethylene insertion became more challenging than the initial ethylene insertion. Additionally, repeated VE insertion also exhibited a quite higher barrier (29.1 kcal/mol), which makes this step unfavorable and can be explained by steric hindrance caused by VE. The computational results were consistent with the experimental data that the copolymer only has in-chain and chain-end -OEt but lacks continuous VE units. Additionally, the barrier for ethylene insertion followed by β-H elimination (24.3 kcal/mol) was higher than that for chain propagation (20.6 kcal/mol), which leads to lower reactivity and decreasing molecular weight. β-OEt elimination and β-H elimination were studied to investigate the chain termination step and the molecular weight of copolymers. β-OEt elimination undergoes much harder than β-H elimination (28.3 kcal/mol vs. 9.4 kcal/mol). Furthermore, the barrier of ethylene insertion followed by β-H elimination (24.3 kcal/mol) is higher than that of chain propagation (20.6 kcal/mol), which indicates that the chain transfer could decrease the molecular weight and reactivity, being consistent with the experimental observation [77]. Also, they explored the effects of different substrates on P atoms, finding that the t-Bu group at phosphine oxide is effective for ethylene copolymerization with VE while the substituent on another phosphorous atom has a minor effect on copolymerization reactivity. Moreover, they also performed DFT calculations for catalyst **B**, finding the barriers for this system are lower than that of **A**, which leads to higher reactivity and incorporation ratios of system **B**. The 1,2-insertion of VE possesses the probability of approx. 69.9% according to the Boltzmann equation, which is much higher than that of system **A** (3.3% probability), indicating that the incorporation of VE may have been improved in system **B**.

**Figure 19:**
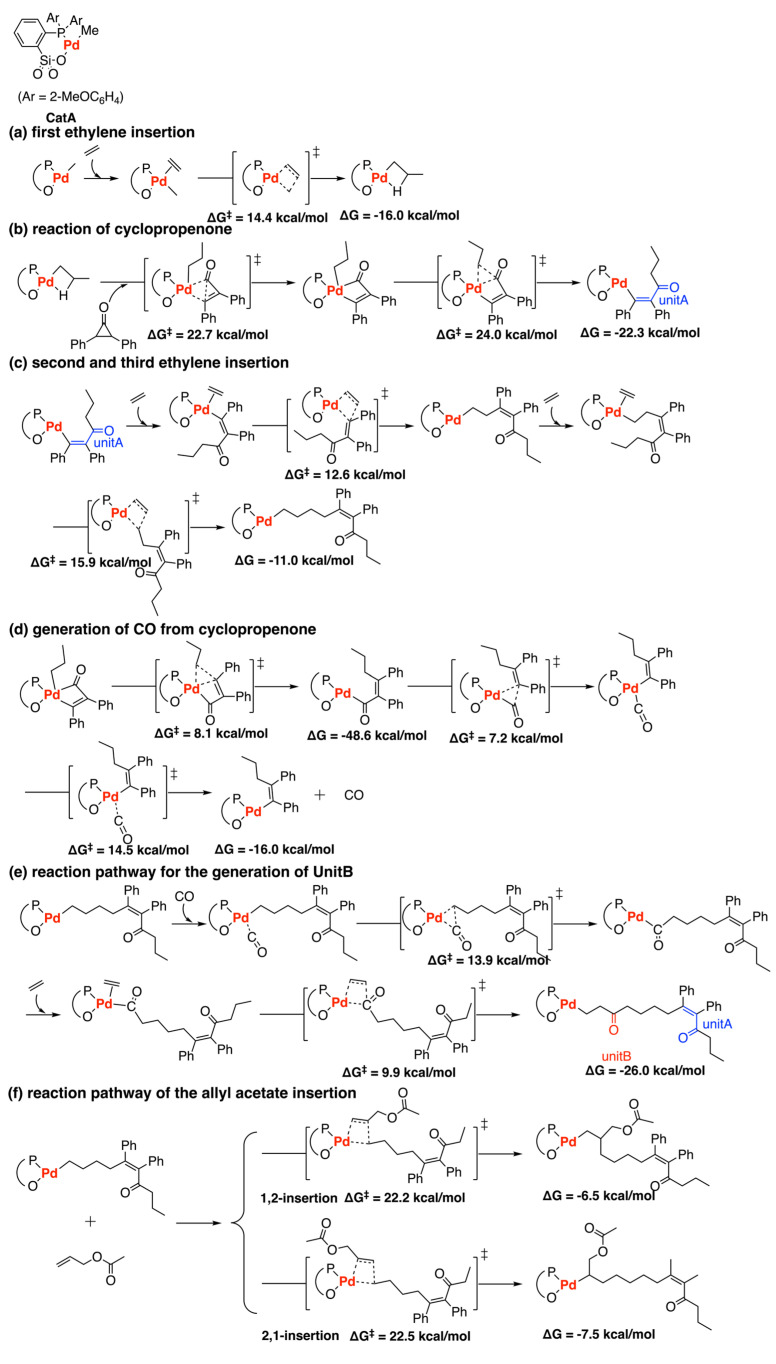
Ethylene copolymerization with cyclopropenone catalyzed by palladium phosphine−sulfonate complex. (**a**) first ethylene insertion; (**b**) reaction of cyclopropenone; (**c**) second and third ethylene insertion; (**d**) generation of CO from cyclopropenone; (**e**) reaction pathway for the generation of UnitB; (**f**) reaction pathway of the allyl acetate insertion.

In 2022, Zhang and coworkers performed DFT calculations to explore the mechanism of ethylene copolymerization with cyclopropenone catalyzed by palladium phosphine-sulfonate complex (Figure 19) [46]. Starting from the chain initiation, *cis*-mode ethylene insertion is the favorable pathway with a barrier of 14.4 kcal/mol, suggesting that this step should be easy to perform. Then, the Pd-alkyl complex could undergo oxidative addition with cyclopropenone to form the intermediate containing a 4-membered ring with a barrier of 22.7 kcal/mol. Afterward, reductive elimination leads to the α,β-unsaturated ketone unit (unitA) (ΔG = −22.3 kcal/mol) with a barrier of 24.0 kcal/mol. The barrier for cyclopropenone insertion is 9.6 kcal/mol higher than that of the initial ethylene insertion reaction but more energy (6.3 kcal/mol) is released in the cyclopropenone insertion than in the first ethylene insertion. The second and third ethylene insertions are feasible kinetically, with a barrier of 12.6 kcal/mol and 15.9 kcal/mol, respectively. Additionally, cyclopropenone could decompose to release CO with the Pd catalyst easily, followed by the incorporation of CO to generate unitB. The energy barrier for unitB generation is 22.7 kcal/mol, while the barrier for unitA generation is 24.0 kcal/mol. Moreover, the copolymerization of ethylene, cyclopropenone, and allyl acetate (AAc) was also investigated. With similar barriers, the cyclopropenone insertion (∆G = −22.3 kcal/mol) releases significantly more energy than the AAc insertion (∆G = −7.5 or −6.5 kcal/mol), indicating that the cyclopropenone introduction reaction is more likely to occur, which is consistent with the experimental results [78]. Moreover, the barriers and the changes of free energy of 1,2-insertion and 2,1-insertion of AAc are similar, showing that they are comparable with each other.

### 3.4. Other Type of Catalysts

**Figure 20:**
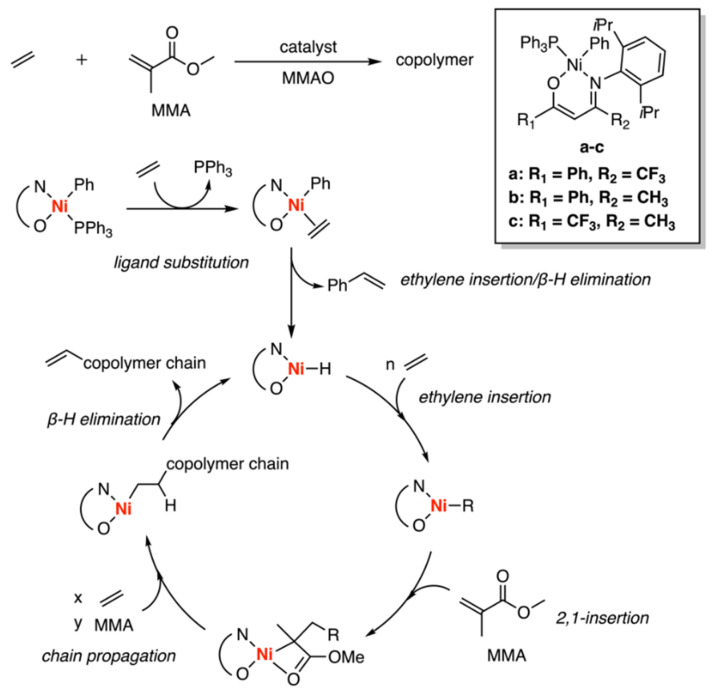
Ethylene copolymerization catalyzed by nickel-based asymmetric β-ketoiminato (N, O) catalysts.

In 2005, Li et al. reported the synthesis of three nickel-based asymmetric β-ketoiminato (N, O) catalysts and modified methylaluminoxane (MMAO) could be utilized for the copolymerization between ethylene and methyl methacrylate (MMA) with excellent yields (>98%), high incorporation (up to 16.7 mol%) and high molecular weight under mild conditions (Figure 20) [47]. Experimental method NMR spectrum revealed a predominance of ethylene-initiated/ethylene-terminated polymers where the methylenoate groups are incorporated into the chain backbone. After the addition of a free radical inhibitor (galvinoxyl), the experiment results remained substantially the same, ruling out the radical mechanism of this reaction. Compared with other relevant reactions [79,80,81,82], the ethylene-enriched copolymer appears the favor of a coordination insertion pathway rather than a radical mechanism. According to their assumed mechanism, the process initiated with the substitution of PPh_3_ by ethylene, followed by the insertion of ethylene and subsequent β-hydrogen elimination, which resulted in the formation of a Ni-H species. Ethylene insertion and MMA 2,1-insertion formed the polyethylene chain as observed in the NMR spectrum. Termination of the ethylene-MMA copolymer chain occurred through β-hydrogen elimination, regenerating the Ni-H species and yielding a vinyl-terminated product.

**Figure 21:**
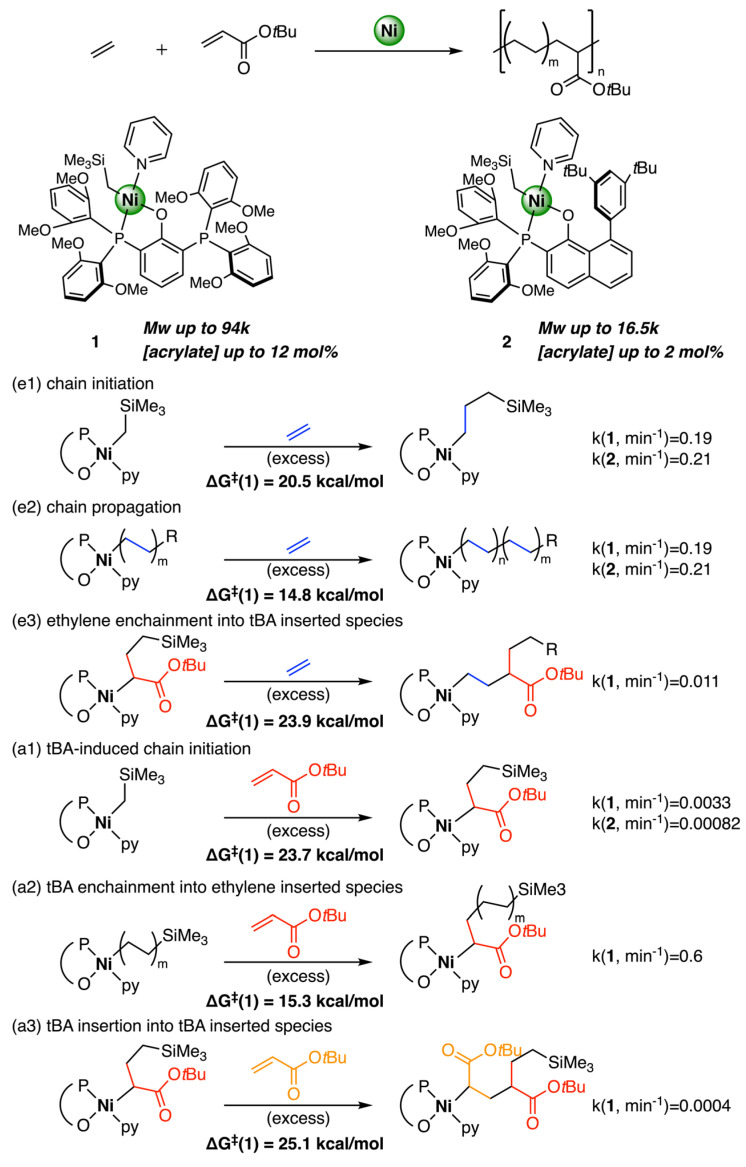
Ethylene copolymerization between ethylene and acrylate catalyzed by nickel−based SHOP catalysts. (**e1**) chain initiation; (**e2**) chain propagation; (**e3**) ethylene enchainment into tBA inserted species; (**a1**) tBA-induced chain initiation; (**a2**) tBA enchainment into ethylene inserted species; (**a3**) tBA insertion into tBA inserted species.

Agapie and Miller utilized experimental kinetics studies (XRD, NMR) and DFT calculations to perform mechanistic insights into ethylene/tert-butyl acrylate (tBA) copolymerization with 2 complexes (Figure 21) [48]. Complex 1 produced copolymers with high molecular weight (Mw = 40,000 g/mol) and high acrylate incorporation (11.95 mol %) while complex 2 produced copolymers with lower molecular weight polymer (Mw = 16,500 g/mol) and lower incorporation of acrylate (1.38%). Kinetics studies revealed initiation (**e1**) and propagation (**e2**) have similar and high rates for both catalysts. For the ethylene and tBA enchainment into ethylene-inserted species (**e2** and **a2**), they have similar barriers (14.8 kcal/mol and 15.3 kcal/mol, respectively) and comparable reaction rates (0.19 min^−1^ and 0.6 min^−1^, respectively), which is a significant aspect of complex 1 that proceed high acrylate incorporation. tBA-induced chain initiation **a1** and the subsequent tBA insertion **a3** were significantly slower compared to the other four insertions. The barriers for **a1** (23.7 kcal/mol) and **a3** (25.1 kcal/mol) were higher than those for the other steps in the copolymerization process, consistent with the copolymer structure. As a result, the rate-determining step of copolymerization is the ethylene reinsertion following tBA insertion, which is approximately one order of magnitude slower than the continuous ethylene insertion.

**Figure 22:**
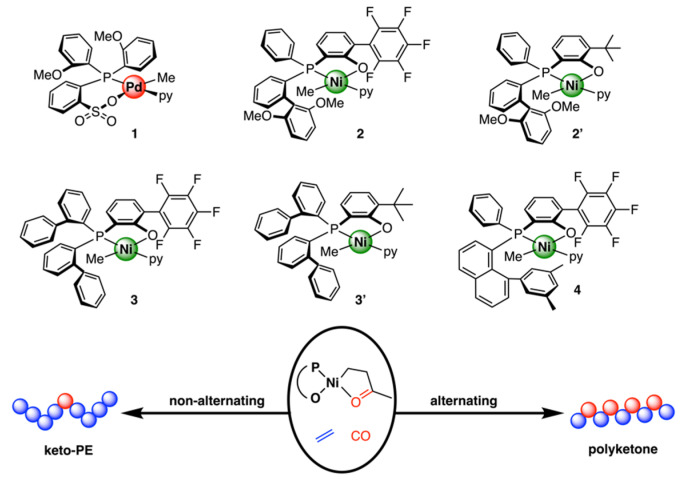
Copolymerization of ethylene and CO catalyzed by Ni(II) SHOP complexes.

In 2022, Mecking, Caporaso, and their coworkers elucidated the mechanism of nonalternating copolymerization of ethylene and CO catalyzed by Ni(II) phosphinephenolate complexes with theoretical DFT study and the experimental study of polymer microstructures (Figure 22) [49]. For the formation of desired nonalternating copolymer, *cis*/*trans* isomerization of the Pd/Ni-alkyl-olefin is the rate-determining step. In addition, the formation of alternating copolymers depends on the barrier for the opening of the six-membered chelate by ethylene binding. The aromatic moiety on the P atom could influence the preference between these two pathways sterically and electronically.

**Figure 23:**
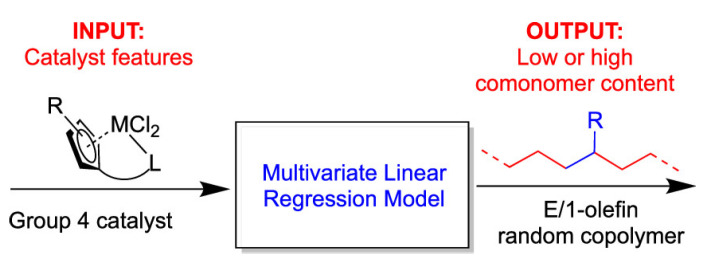
MLR model used to predict the comonomer content [51].

Cavallo and coworkers reported a Web application, namely SambVca, aimed at analyzing the catalytic pockets of metal complexes, employing a topographic steric descriptor known as percent buried volume (%V_Bur_) that is suitable for the design of transition metal catalysts [50]. Furthermore, they conducted research in which they utilized a combination of Density Functional Theory (DFT) and multivariate linear regression (MLR) to predict the comonomer incorporation rate in the copolymerization of ethylene and 1-olefins with group 4 catalysts [51] (Figure 23). %V_Bur_, average chloride charge, and reduction potential were identified as crucial descriptors that yielded the best-fit MLR equation. Additionally, their investigations extended to the prediction of the enantioselectivity of propene polymerization. Burid volume of quadrants and octants, namely %*V*_BQ_ and %*V*_BO_ were used as new descriptors to predict the enantioselectivity [52].

**Figure 24:**
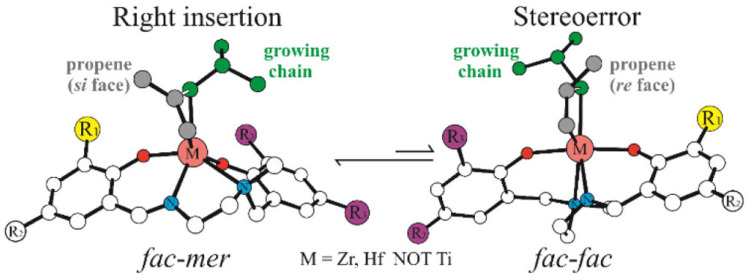
DFT optimized conformational change for propene polymerization [53].

In addition to the aforementioned examples, Talarico and colleagues focused on the theoretical study on the mechanisms of the olefin polymerization catalyzed by group 4 metals, namely Titanium (Ti), Zirconium (Zr), and Hafnium (Hf) [53,54,55]. Steric properties and stereoselectivity are the key points of this study. In 2022, DFT calculations in conjunction with the Activation Strain Model (ASM) were employed to explore the conformational changes during the process of propene polymerization catalyzed by *C*_1_-symmetric salalen m systems [53] (Figure 24). Their research presented distinct catalytic cycles proposed for Ti and Zr/Hf salalen systems, shedding light on the lower stereoselectivities obtained with Zr and Hf. Subsequently, %V_Bur_ and natural energy decomposition analysis (NEDA) were also utilized to delve into the chain shuttling process during the polymerizations, offering detailed insights into the coordination geometries of the reactions [54]. Furthermore, they applied these same methods to investigate the origins of stereoselectivity in propene polymerization facilitated by pyridylamido-type non-metallocene systems [55], revealing how steric and electronic effects influence the outcomes of the reactions.

In 2021, Bahri-Laleh and coworkers reported a DFT study on the ethylene trimerization catalyzed by Ti-based half-sandwich metallocene complexes in the presence of methyl aluminoxane (MAO) [56], proposing a general mechanism for ethylene trimerization via metallacycle intermediates and rationalizing the varying reactivities observed in the different Ti complexes. They also used a combination of DFT calculations and %V_Bur_ to explore the cocatalyst tyle effect on the Ziegler-Natta catalyzed ethylene polymerizations, explaining why triisobutyl aluminum (TIBA) leads to lower activity compared to triethyl aluminum (TEA). Additionally, their investigation extended to exploring the impact of cocatalyst types on Ziegler-Natta catalyzed ethylene polymerizations [57]. Through a combination of DFT calculations and the percent buried volume (%V_Bur_) descriptor, they shed light on why triisobutyl aluminum (TIBA) results in lower activity compared to triethyl aluminum (TEA). This work contributes to a deeper understanding of the effects of different cocatalysts on polymerization reactions.

## 4. Summary and Perspective

In this review, the latest developments in mechanistic studies of ethylene copolymerization with polar monomers catalyzed by late-transition-metal complexes were comprehensively reviewed. The incorporation of a minor proportion of polar monomers into polyolefins could introduce additional physical and chemical properties depending on the functional groups. A variety of fundamental polar vinyl monomers, including methyl acrylate (MA), methyl methacrylate (MMA), vinyl acetate (VA), vinylalkoxysilanes, acrylonitrile (AN), allyl ethyl ether (AEE), diallyl ether (DAE), vinyl ether (VE), cyclopropenone, and tert-butyl acylate (tBA), have been studied for successful incorporation into polyethylene. Experimental methods such as EPR, low-temperature NMR, and XRD were employed for the mechanistic investigation, leading to the identification of resting states and the estimation of kinetic data. Computational methods such as DFT studies and molecular dynamics (MD) simulations were applied to map out reaction pathways. The combination of experimental and computational methods significantly enhances the understanding of ethylene copolymerization reactions catalyzed by Pd- and Ni-based complexes, including the Brookhart-type, Grubbs-type, Drent-type, and other similar complexes. The mechanistic study mainly focused on the fundamental steps including ligand substitution, changes in binding modes, ethylene insertion, polar monomer insertion, chelate-opening, β-H elimination, and so on. From the results above, factors that control the catalytic activity, molecular weight, comonomer incorporation ratios, and branch content emerged. These include steric repulsions between ligands and monomers, electronic effects originating from both ligands and monomers, etc.

## Data Availability

This is a review article and hence no additional data are available.

## References

[1] Annual Production of Plastics Worldwide from 1950 to 2021. https://www.statista.com/statistics/282732/global-production-of-plastics-since-1950/.

[2] Dong J.Y., Hu Y. (2006). Design and synthesis of structurally well-defined functional polyolefins via transition metal-mediated olefin polymerization chemistry. Coord. Chem. Rev..

[3] Rünzi T., Mecking S. (2014). Saturated Polar-Substituted Polyethylene Elastomers from Insertion Polymerization. Adv. Funct. Mater..

[4] Dai S., Chen C. (2018). Palladium-Catalyzed Direct Synthesis of Various Branched, Carboxylic Acid-Functionalized Polyolefins: Characterization, Derivatization, and Properties. Macromolecules.

[5] Na Y., Dai S., Chen C. (2018). Direct Synthesis of Polar-Functionalized Linear Low-Density Polyethylene (LLDPE) and Low-Density Polyethylene (LDPE). Macromolecules.

[6] Sui X., Hong C., Pang W., Chen C. (2017). Unsymmetrical a-diimine palladium catalysts and their properties in olefin (co)polymerization. Mater. Chem. Front..

[7] Boaen N.K., Hillmyer M.A. (2005). Post-polymerization functionalization of polyolefins. Chem. Soc. Rev..

[8] Lehman S.E., Wagener K.B., Baugh L.S., Rucker S.P., Schulz D.N., Varma-Nair M., Berluche E. (2007). Linear Copolymers of Ethylene and Polar Vinyl Monomers via Olefin Metathesis-Hydrogenation: Synthesis, Characterization, and Comparison to Branched Analogues. Macromolecules.

[9] Yang H., Islam M., Budde C., Rowan S.J. (2003). Ring-Opening Metathesis Polymerization as a Route to Controlled Copolymers of Ethylene and Polar Monomers: Synthesis of Ethylene–Vinyl Chloride-Like Copolymers. J. Polym. Sci. Part A Polym. Chem..

[10] Hillmyer M.A., Laredo W.R., Grubbs R.H. (1995). Ring-Opening Metathesis Polymerization of Functionalized Cyclooctenes by a Ruthenium-Based Metathesis Catalyst?. Macromolecules.

[11] Mutlu H., de Espinosa L.M., Meier M.R. (2011). Acyclic diene metathesis: A versatile tool for the construction of defined polymer architectures. Chem. Soc. Rev..

[12] Boz E., Nemeth A.J., Ghiviriga I., Jeon K., Alamo R.G., Wagener K.B. (2007). Precision Ethylene/Vinyl Chloride Polymers via Condensation Polymerization. Macromolecules.

[13] Boz E., Nemeth A.J., Alamo R.G., Wagener K.B. (2007). Precision Ethylene/Vinyl Bromide Polymers. Adv. Synth. Catal..

[14] Boz E., Wagener K.B., Ghosal A., Fu R., Alamo R.G. (2006). Synthesis and Crystallization of Precision ADMET Polyolefins Containing Halogens. Macromolecules.

[15] Nakamura A., Ito S., Nozaki K. (2009). Coordination-Insertion Copolymerization of Fundamental Polar Monomers. Chem. Rev..

[16] Chen J., Gao Y., Marks T.J. (2020). Early Transition Metal Catalysis for Olefin-Polar Monomer Copolymerization. Angew. Chem. Int. Ed..

[17] Johnson L.K., Killian C.M., Brookhart M. (1995). New Pd(II)- and Ni(II)-Based Catalysts for Polymerization of Ethylene and α-Olefins. J. Am. Chem. Soc..

[18] Younkin T.R., Connor E.F., Henderson J.I., Friedrich S.K., Grubbs R.H., Bansleben D.A. (2000). Neutral, Single-Component Nickel (II) Polyolefin Catalysts that Tolerate Heteroatoms. Science.

[19] Drent E., van Dijk R., van Ginkel R., van Oort B., Pugh R.I. (2002). Palladium catalysed copolymerisation of ethene with alkylacrylates: Polar comonomer built into the linear polymer chain. Chem. Commun..

[20] Wucher P., Goldbach V., Mecking S. (2013). Electronic Influences in Phosphinesulfonato Palladium(II) Polymerization Catalysts. Organometallics.

[21] Chen M., Chen C. (2017). Rational Design of High-Performance Phosphine Sulfonate Nickel Catalysts for Ethylene Polymerization and Copolymerization with Polar Monomers. ACS Catal..

[22] Carrow B.P., Nozaki K. (2012). Synthesis of Functional Polyolefins Using Cationic Bisphosphine Monoxide-Palladium Complexes. J. Am. Chem. Soc..

[23] Mitsushige Y., Yasuda H., Carrow B.P., Ito S., Kobayashi M., Tayano T., Watanabe Y., Okuno Y., Hayashi S., Kuroda J. (2018). Methylene-Bridged Bisphosphine Monoxide Ligands for Palladium-Catalyzed Copolymerization of Ethylene and Polar Monomers. ACS Macro Lett..

[24] Xin B.S., Sato N., Tanna A., Oishi Y., Konishi Y., Shimizu F. (2017). Nickel-Catalyzed Copolymerization of Ethylene and Alkyl Acrylates. J. Am. Chem. Soc..

[25] Chen M., Chen C. (2018). A Versatile Ligand Platform for Palladium- and Nickel-Catalyzed Ethylene Copolymerization with Polar Monomers. Angew. Chem. Int. Ed..

[26] Michalak A., Ziegler T. (2001). DFT Studies on the Copolymerization of R-Olefins with Polar Monomers: Ethylene-Methyl Acrylate Copolymerization Catalyzed by a Pd-Based Diimine Catalyst. J. Am. Chem. Soc..

[27] Michalak A., Ziegler T. (2001). DFT Studies on the Copolymerization of α-Olefins with Polar Monomers: Comonomer Binding by Nickel- and Palladium-Based Catalysts with Brookhart and Grubbs Ligands. Organometallics.

[28] Deubel D.V., Ziegler T. (2002). DFT Study of Olefin versus Nitrogen Bonding in the Coordination of Nitrogen-Containing Polar Monomers to Diimine and Salicylaldiminato Nickel(II) and Palladium(II) Complexes. Implications for Copolymerization of Olefins with Nitrogen-Containing Polar Monomers. Organometallics.

[29] Michalak A., Ziegler T. (2003). A Comparison of Ni- and Pd-Diimine Complexes as Catalysts for Ethylene/Methyl Acrylate Copolymerization. A Static and Dynamic Density Functional Theory Study. Organometallics.

[30] Szabo M.J., Jordan R.F., Michalak A., Piers W.E., Weiss T., Yang S.Y., Ziegler T. (2004). Polar Copolymerization by a Palladium-Diimine-Based Catalyst. Influence of the Catalyst Charge and Polar Substituent on Catalyst Poisoning and Polymerization Activity. A Density Functional Theory Study. Organometallics.

[31] Williams B.S., Leatherman M.D., White P.S., Brookhart M. (2005). Reactions of Vinyl Acetate and Vinyl Trifluoroacetate with Cationic Diimine Pd(II) and Ni(II) Alkyl Complexes: Identification of Problems Connected with Copolymerizations of These Monomers with Ethylene. J. Am. Chem. Soc..

[32] Chen Z., Liu W., Daugulis O., Brookhart M. (2016). Mechanistic Studies of Pd(II)-Catalyzed Copolymerization of Ethylene and Vinylalkoxysilanes: Evidence for a β Silyl Elimination Chain Transfer Mechanism. J. Am. Chem. Soc..

[33] Li M., Wang X., Luo Y., Chen C. (2017). A Second-Coordination-Sphere Strategy to Modulate Nickel- and Palladium-Catalyzed Olefin Polymerization and Copolymerization. Angew. Chem. Int. Ed..

[34] Michalak A., Ziegler T. (2000). DFT Studies on Substituent Effects in Palladium-Catalyzed Olefin Polymerization. Organometallics.

[35] Michalak A., Ziegler T. (2003). Polymerization of Ethylene Catalyzed by a Nickel(+2) Anilinotropone-Based Catalyst: DFT and Stochastic Studies on the Elementary Reactions and the Mechanism of Polyethylene Branching. Organometallics.

[36] Waltman A.W., Younkin T.R., Grubbs R.H. (2004). Insights into the Deactivation of Neutral Nickel Ethylene Polymerization Catalysts in the Presence of Functionalized Olefins. Organometallics.

[37] Berkefeld A., Drexler M., Moller H.M., Mecking S. (2009). Mechanistic Insights on the Copolymerization of Polar Vinyl Monomers with Neutral Ni(II) Catalysts. J. Am. Chem. Soc..

[38] Berkefeld A., Mecking S. (2009). Deactivation Pathways of Neutral Ni(II) Polymerization Catalysts. J. Am. Chem. Soc..

[39] Olscher F., Schnetmann I.G., Monteil V., Mecking S. (2015). Role of Radical Species in Salicylaldiminato Ni(II) Mediated Polymer Chain Growth: A Case Study for the Migratory Insertion Polymerization of Ethylene in the Presence of Methyl Methacrylate. J. Am. Chem. Soc..

[40] Haras A., Anderson G.W., Michalak A., Rieger B., Ziegler T. (2006). Computational Insight into Catalytic Control of Poly(ethylene-methyl acrylate) Topology. Organometallics.

[41] Noda S., Nakamura A., Kochi T., Chung L.W., Morokuma K., Nozaki K. (2009). Mechanistic Studies on the Formation of Linear Polyethylene Chain Catalyzed by Palladium Phosphine-Sulfonate Complexes: Experiment and Theoretical Studies. J. Am. Chem. Soc..

[42] Nozaki K., Kusumoto S., Noda S., Kochi T., Chung L.W., Morokuma K. (2010). Why Did Incorporation of Acrylonitrile to a Linear Polyethylene Become Possible? Comparison of Phosphine-Sulfonate Ligand with Diphosphine and Imine-Phenolate Ligands in the Pd-Catalyzed Ethylene/Acrylonitrile Copolymerization. J. Am. Chem. Soc..

[43] Wimmer F.P., Caporaso L., Cavallo L., Mecking S., Falivene L. (2018). Mechanism of Insertion Polymerization of Allyl Ethers. Macromolecules.

[44] Sun J., Chen M., Luo G., Chen C., Luo Y. (2019). Diphosphazane-monoxide and Phosphine-sulfonate Palladium Catalyzed Ethylene Copolymerization with Polar Monomers: A Computational Study. Organometallics.

[45] Mehmood A., Xu X., Raza W., Kim K.H., Luo Y. (2020). Mechanistic Studies for Palladium Catalyzed Copolymerization of Ethylene with Vinyl Ethers, Mechanistic Studies for Palladium Catalyzed Copolymerization of Ethylene with Vinyl Ethers. Polymers.

[46] Zhang C., Yu S., Wang F., Wang F., Cao J., Zheng H., Chen X., Ren A. (2022). Density Functional Theory Analysis of the Copolymerization of Cyclopropenone with Ethylene Using a Palladium Catalyst. Polymers.

[47] Li X., Li Y., Li Y., Chen Y., Hu N. (2005). Copolymerization of Ethylene with Methyl Methacrylate with Neutral Nickel(II) Complexes Bearing β-Ketoiminato Chelate Ligands. Organometallics.

[48] Xiong S., Shoshani M.M., Zhang X., Spinney H.A., Nett A.J., Henderson B.S., Miller T.F., Agapie T. (2021). Efficient Copolymerization of Acrylate and Ethylene with Neutral P, O-Chelated Nickel Catalysts: Mechanistic Investigations of Monomer Insertion and Chelate Formation. J. Am. Chem. Soc..

[49] Voccia M., Odenwald L., Baur M., Lin F., Falivene L., Mecking S., Caporaso L. (2022). Mechanistic Insights into Ni(II)-Catalyzed Nonalternating Ethylene-Carbon Monoxide Copolymerization. J. Am. Chem. Soc..

[50] Falivene L., Credendino R., Poater A., Petta A., Serra L., Oliva R., Scarano V., Cavallo L. (2016). SambVca 2. A Web Tool for Analyzing Catalytic Pockets with Topographic Steric Maps. Organometallics.

[51] Maity B., Cao Z., Kumawat J., Gupta V., Cavallo L. (2021). A Multivariate Linear Regression Approach to Predict Ethene/1- Olefin Copolymerization Statistics Promoted by Group 4 Catalysts. ACS Catal..

[52] Antinucci G., Dereli B., Vittoria A., Budzelaar P.M., Cipullo R., Goryunov G.P., Kulyabin P.S., Uborsky D.V., Cavallo L., Ehm C. (2022). Selection of Low-Dimensional 3-D Geometric Descriptors for Accurate Enantioselectivity Prediction. ACS Catal..

[53] Romano E., Budzelaar P.M., Rosa C.D., Talarico G. (2022). Unconventional Stereoerror Formation Mechanisms in Nonmetallocene Propene Polymerization Systems Revealed by DFT Calculations. J. Phys. Chem. A.

[54] Zunino R., Luise D., Barone V., Rosa C.D., Talarico G. (2023). Reversible trans-alkylation mechanisms at homogeneous catalytic systems investigated by a combined DFT and ASM-NEDA approach. Mol. Catal..

[55] D’Anania O., Rosa C.D., Talarico G. (2023). A Computational Evaluation of the Steric and Electronic Contributions in Stereoselective Olefin Polymerization with Pyridylamido-Type Catalysts. Molecules.

[56] Gharajedaghi S., Mohamadnia Z., Ahmadi E., Marefat M., Pareras G., Simon S., Poater A., Bahri-Laleh N. (2021). Experimental and DFT study on titanium-based half-sandwich metallocene catalysts and their application for production of 1-hexene from ethylene. Mol. Catal..

[57] Masoori M., Nekoomanesh M., Posada-Pérez S., Rashedi R., Bahri-Laleh N. (2022). Exploring cocatalyst type effect on the Ziegler–Natta catalyzed ethylene polymerizations: Experimental and DFT studies. J. Polym. Res..

[58] Cohen A.J., Mori-Sanchez P., Yang W. (2008). Insights into current limitations of density functional theory. Science.

[59] Cramer C.J., Truhlar D.G. (2009). Density functional theory for transition metals and transition metal chemistry. Phys. Chem. Chem. Phys..

[60] Simon L., Goodman J.M. (2011). How reliable are DFT transition structures? Comparison of GGA, hybrid-meta-GGA and meta-GGA functionals. Org. Biomol. Chem..

[61] Minenkov Y., Singstad A., Occhipinti G., Jensen V.R. (2012). The accuracy of DFT-optimized geometries of functional transition metal compounds: A validation study of catalysts for olefin metathesis and other reactions in the homogeneous phase. Dalton Trans..

[62] Cohen A.J., Mori-Sanchez P., Yang W. (2012). Challenges for density functional theory. Chem. Rev..

[63] Burke K. (2012). Perspective on density functional theory. J. Chem. Phys..

[64] Becke A.D. (2014). Perspective: Fifty years of density-functional theory in chemical physics. J. Chem. Phys..

[65] Jones R.O. (2015). Density functional theory: Its origins, rise to prominence, and future. Rev. Mod. Phys..

[66] Pribram-Jones A., Gross D.A., Burke K. (2015). DFT: A theory full of holes?. Annu. Rev. Phys. Chem..

[67] Yu H.S., Li S.L., Truhlar D.G. (2016). Perspective: Kohn-Sham density functional theory descending a staircase. J. Chem. Phys..

[68] TeVelde G., Bickelhaupt F.M., Baerends E.J., Fonseca Guerra C., Van Gisbergen S.J.A., Snijders J.G., Ziegler T.J. (2001). Chemistry with ADF. Comput. Chem..

[69] Frisch M.J., Trucks G.W., Schlegel H.B., Scuseria G.E., Robb M.A., Cheeseman J.R., Montgomery J.A., Vreven T., Kudin K.N., Burant J.C. (2003). Gaussian 03.

[70] Frisch M.J., Trucks G.W., Schlegel H.B., Scuseria G.E., Robb M.A., Cheeseman J.R., Scalmani G., Barone V., Mennucci B., Petersson G.A. (2009). Gaussian 09.

[71] Frisch M.J., Trucks G.W., Schlegel H.B., Scuseria G.E., Robb M.A., Cheeseman J.R., Scalmani G., Barone V., Petersson G.A., Nakatsuji H. (2016). Gaussian 16.

[72] Hicks F.A., Brookhart M. (2001). A Highly Active Anilinotropone-Based Neutral Nickel(II) Catalyst for Ethylene Polymerization. Organometallics.

[73] Chan M.W., Deng L., Ziegler T. (2000). Density Functional Study of Neutral Salicylaldiminato Nickel(II) Complexes as Olefin Polymerization Catalysts. Organometallics.

[74] Kochi T., Noda S., Yoshimura K., Nozaki K. (2007). Formation of Linear Copolymers of Ethylene and Acrylonitrile Catalyzed by Phosphine Sulfonate Palladium Complexes. J. Am. Chem. Soc..

[75] Jian Z., Mecking S. (2015). Insertion Homo- and Copolymerization of Diallyl Ether. Angew. Chem. Int. Ed..

[76] Runzi T., Guironnet D., Schnetmann I.G., Mecking S. (2010). Reactivity of Methacrylates in Insertion Polymerization. J. Am. Chem. Soc..

[77] Luo S., Vela J., Lief G.R., Jordan R.F. (2007). Copolymerization of Ethylene and Alkyl Vinyl Ethers by a (Phosphinesulfonate)PdMe Catalyst. J. Am. Chem. Soc..

[78] Wang X., Seidel F.W., Nozaki K. (2019). Synthesis of Polyethylene with in-chain α,β-Unsaturated Ketone and Isolated Ketone Units by Pd-catalyzed Ring-opening Copolymerization of Cyclopropenones with Ethylene. Angew. Chem. Int. Ed..

[79] Johnson L.K., Mecking S., Brookhart M. (1996). Copolymerization of Ethylene and Propylene with Functionalized Vinyl Monomers by Palladium(II) Catalysts. J. Am. Chem. Soc..

[80] Mecking S., Johnson L.K., Wang L., Brookhart M. (1998). Mechanistic Studies of the Palladium-Catalyzed Copolymerization of Ethylene and R-Olefins with Methyl Acrylate. J. Am. Chem. Soc..

[81] Tian G., Boone H.W., Novak B.M. (2001). Neutral Palladium Complexes as Catalysts for Olefin-Methyl Acrylate Copolymerization: A Cautionary Tale. Macromolecules.

[82] Elia C., Barad S.E., Sen A. (2002). Palladium-Based System for the Polymerization of Acrylates. Scope and Mechanism. Organometallics.

